# Pancreatic ACTH Hypersecretion and Pituitary Macroadenoma

**DOI:** 10.1210/jcemcr/luad007

**Published:** 2023-02-01

**Authors:** Chiara M Bettale, Jason W Allen, Zaid K Mahdi, Adriana G Ioachimescu

**Affiliations:** Emory University School of Medicine, Atlanta, GA 30322, USA; Department of Radiology and Imaging Services, Emory University School of Medicine, Atlanta, GA 30322, USA; Department of Neurology, Emory University School of Medicine, Atlanta, GA 30322, USA; Department of Pathology, Emory University School of Medicine, Atlanta, GA 30322, USA; Department of Medicine, Division of Endocrinology, Metabolism and Lipids, Emory University School of Medicine, Atlanta, GA 30322, USA; Department of Neurosurgery, Emory University School of Medicine, Atlanta, GA 30322, USA

**Keywords:** Cushing syndrome, ectopic ACTH syndrome, neuroendocrine tumor, pancreatic neuroendocrine neoplasm, pituitary adenoma

## Abstract

A 55-year-old woman admitted for hypertensive emergency and myocardial infarction reported weight gain, muscle weakness, easy bruising, and recent-onset diabetes in the past 3 to 12 months. Urinary and salivary cortisol and adrenocorticotropin hormone (ACTH) levels were elevated. Pituitary imaging detected a macroadenoma. ACTH and cortisol did not increase after corticotropin-releasing hormone administration. Imaging revealed a large pancreatic mass. Pathology indicated a well-differentiated World Health Organization (WHO) grade 2 distal pancreatic neuroendocrine neoplasm which stained for ACTH by immunohistochemistry. Postoperatively, Cushing manifestations resolved, ACTH and cortisol levels became low, and patient required hydrocortisone replacement for 7 months. During the 3.5 years of follow-up, the pituitary macroadenoma size remained stable and pituitary hormone axes other than ACTH remained normal. This extremely rare case of ectopic ACTH-secreting pancreatic neuroendocrine tumor coexisting with a nonfunctioning pituitary macroadenoma illustrates the importance of dynamic endocrine testing in Cushing syndrome.

## Introduction

We present a case of adrenocorticotropin hormone (ACTH)-dependent Cushing syndrome (CS) with coexistence of a pituitary macroadenoma and a pancreatic neuroendocrine neoplasm (pNEN). Pancreatic ACTH secretion was confirmed by immunohistochemistry, and the hypercortisolism resolved after pancreatic surgery. To our knowledge, this is the first reported case of ACTH-secreting pNEN coexistent with a nonfunctioning pituitary macroadenoma.

Diagnosis and management of ACTH-depending CS can be challenging, especially when the pituitary or ectopic tumor is too small to be detected on imaging [1]. In this case, however, the patient had a pituitary macroadenoma. Its removal would have delayed the diagnosis of the pNEN and prolonged the duration of hypercortisolism.

Pituitary tumors represent the most common cause of CS, with microadenomas much more frequent than macroadenomas. Non-pituitary ACTH-producing neuroendocrine neoplasms (NENs) represent approximately 10% of CS cases. Among ectopic tumors causing CS, pulmonary carcinoids are most prevalent (>25%), followed by small cell lung cancers (∼20%), thymic NEN (11%), pancreatic NEN (8%), medullary thyroid carcinomas (6%), and pheochromocytomas (5%). In 20% of cases of ectopic CS, the source remains unknown [2].

Incidence of pNENs is 1 to 2 per 100 000/year and the majority are nonfunctional; among functional pNEN, most frequent are insulinomas and glucagonomas. ACTH-producing pNEN are very rare (7%). Some patients have extensive distant metastases at presentation, which precludes postoperative remission of hypercortisolism [3].

## Case Presentation

A 55-year-old woman with newly diagnosed type 2 diabetes and hypertension presented to the emergency room with chest pain, dizziness and shortness of breath. She was diagnosed with hypertensive urgency and non-ST elevation myocardial infarction, which were medically treated. Patient reported muscle weakness, fatigue, and uncontrolled blood pressure despite increasing her medications in the last year. She endorsed a 30-kg weight gain over the past 3 months; also leg edema, easy bruising, striae, and hair thinning 1 month prior to presentation. Family history was remarkable for pancreatic tumor in her grandmother.

During hospitalization, laboratory studies indicated hypokalemia (2.9 mEq/L or 2.9 mmol/L), and very high serum cortisol, plasma ACTH, and 24-hour urine cortisol levels (Table 1). Non-contrast brain MRI indicated a pituitary adenoma and the pulmonary CT angiogram was remarkable for bilateral adrenal enlargement with a 1.1-cm left adrenal nodule.

**Table 1.** Preoperative workup for hypercortisolism

Test	Inpatient	Outpatient
Serum cortisol	53.9 μg/dL (1487.6 nmol/L)	27.5 μg/dL (759 nmol/L)
Adrenocorticotropic hormone	450 pg/mL (99 pmol/L)	307 pg/mL (67.5 pmol/L)
24-hour urinary free cortisol	>3500 μg/day (>9660 nmol/day)	499.6; 662.8 μg/day (1378.9; 1829.3 nmol/day)
Serum cortisol after 1-mg dexamethasone		18.5 μg/dL (510.6 nmol/L)
Serum cortisol after 8-mg dexamethasone		28.8*^a^* μg/dL (794.9 nmol/L)

Normal ranges: AM serum cortisol 6.2 to 19.4 μg/dL (171.1-535.4 nmol/L), ACTH 7.2 to 63.3 pg/mL (1584-13 926 pmol/L), 24-hour urinary free cortisol 0 to 50 μg/24 hour (0-138 nmol/day).

a Dexamethasone level 2250 ng/dL (57 330 pmol/L).

Upon outpatient evaluation after discharge, blood pressure was 139/81, pulse 100, weight 100 kg, BMI 39.7 kg/m^2^. Physical exam was significant for rounded face, supraclavicular fat pads, dorsocervical fullness, discolored abdominal and thigh striae, and proximal weakness. Medications included aspirin 325 mg daily, atorvastatin 40 mg daily, metformin 500 mg twice a day, amlodipine 10 mg daily, benazepril 40 mg daily, carvedilol 12.5 mg twice a day, terazosin 2 mg nightly, spironolactone 100 mg daily, KCl 10 mEq twice a day, omeprazole 20 mg daily, meloxicam 7.5 mg twice a day, doxepin 10 mg as needed for insomnia, and nitroglycerin 0.4 mg sublingual as need for chest pain.

## Diagnostic Assessment

**Figure 1.**
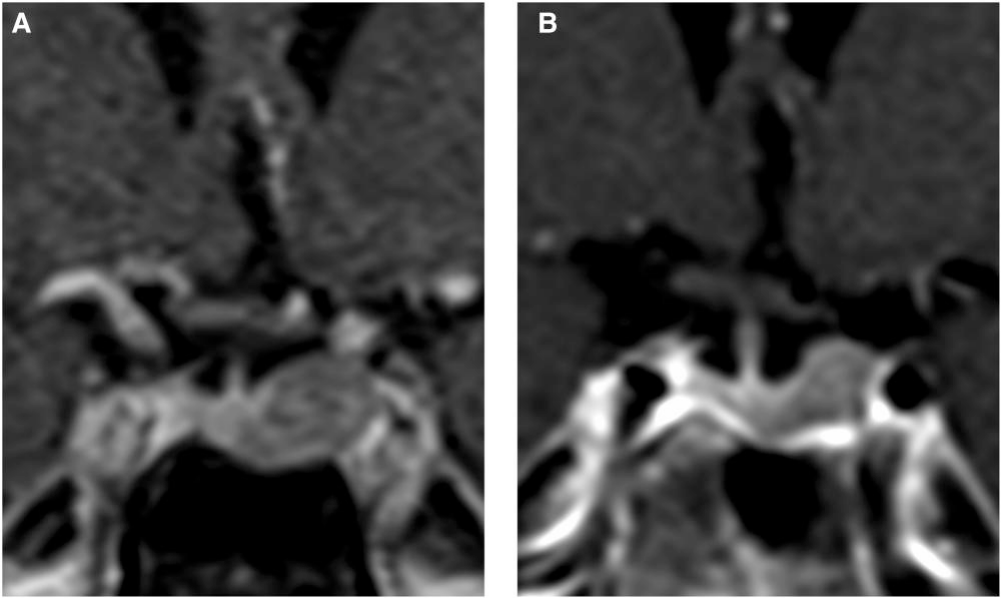
(A) Contrast-enhanced coronal T1-weighted image centered on the sella demonstrates a hypo-enhancing lesion in the left aspect of the sella measuring 1.2 × 1.3 × 0.9 cm (AP, lateral, craniocaudal dimensions) with extension to the lateral intracarotid line (Knosp grade 2, not shown). Mild suprasellar extension without encroachment upon the optic chiasm or prechiasmatic optic nerve. (B) Contrast-enhanced coronal T1-weighed image centered on the sella obtained 3 years and 3 months later demonstrates mild interval decrease in the size of the hypo-enhancing lesion in the left sella, which now measures 1.2 × 1.0 × 0.7 cm (AP, lateral, craniocaudal dimensions), again with extension to the lateral intracarotid line (Knosp grade 2, not shown). Persistent mild suprasellar extension.

Laboratory studies were repeated in the outpatient setting and confirmed ACTH-dependent hypercortisolism (Table 1). Pituitary MRI with and without contrast showed a left sellar mass compatible with a pituitary macroadenoma measuring 1.0 × 1.3 × 0.9 cm (Fig. 1). Pituitary laboratory tests other than cortisol and ACTH were unremarkable.

We performed dynamic tests because of presentation with hypokalemia and rapidly evolving clinical manifestations, as well as very high levels of ACTH (5 × above upper normal limit [ULN]) and urinary cortisol (10 × ULN). There was no decrease of serum cortisol after high-dose dexamethasone and no increase in ACTH or cortisol levels after corticotropin-releasing hormone (CRH) administration (Table 2). Chromogranin A was high (10 400 ng/mL or μg/L; normal: 0-103). Calcitonin (undetectable; normal < 10 pg/mL or ng/L), gastrin (34 pg/mL or ng/L; normal: 0-100), plasma free normetanephrine (0.58 pg/mL or 3.17 pmol/L; normal: 0-0.89 pg/mL or 0-4.86 pmol/L) and metanephrines (0.11 pg/mL or 0.56 pmol/L; normal: 0-0.49 pg/mL or 0-2.48 pmol/L). Calcium levels were normal.

**Table 2.** Dexamethasone combined with CRH test results

	−15 minutes	0 minutes	15 minutes	30 minutes	45 minutes	60 minutes
Cortisol	27.1 μg/dL (748 nmol/L)	28.2 μg/dL (778.3 nmol/L)	26.1 μg/dL (720.4 nmol/L)	27.6 μg/dL (761.8 nmol/L)	26.3 μg/dL (725.9 nmol/L)	26.6 μg/dL (734.2 nmol/L)
ACTH	262 pg/mL (57.6 pmol/L)	276 pg/mL (60.7 pmol/L)	230 pg/mL (50.6 pmol/L)	262 pg/mL (57.6 pmol/L)	249 pg/mL (54.8 pmol/L)	278 pg/mL (61.2 pmol/L)
Dexamethasone level	491 ng/dL (12 510.7 pmol/L)					

Protocol highlights:

Dexamethasone 0.5 mg orally every 6 hours was started at 12 Pm (8 doses). Using an indwelling peripheral IV line, blood was drawn at 8 Am (2 hours after last dexamethasone dose) and 8:15 Am. Then, corticorelin 100 μg was administered intravenously. Repeated blood collections were done 15, 30, 45, and 60 minutes later.

**Figure 2.**
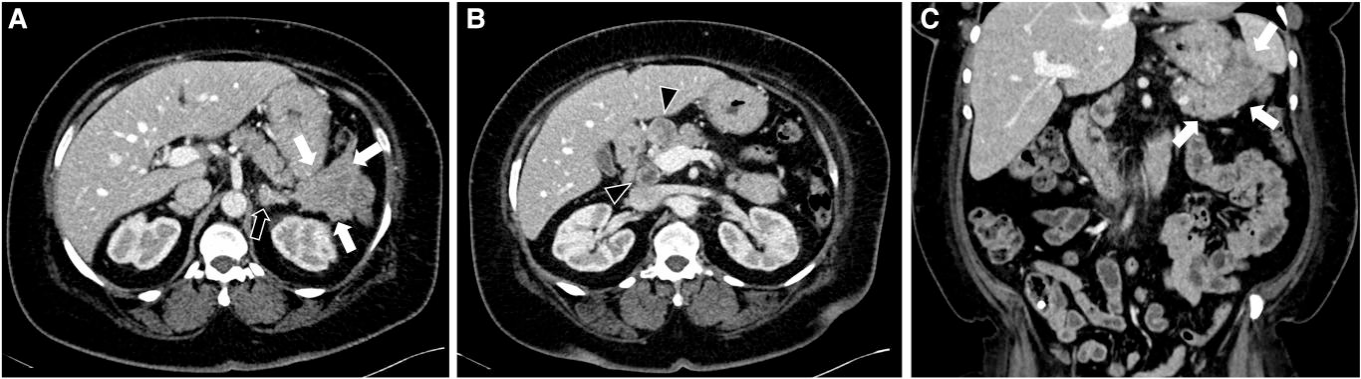
Contrast-enhanced abdomen CT in the axial (A, B) and coronal (C) planes demonstrates a lobulated, heterogeneously enhancing mass arising from the tail of the pancreas (white arrows, 2A, 2C) measuring 5.9 × 5.1 × 6.1 cm (AP, lateral, craniocaudal dimensions). Mass obliterates the splenic vein (not shown). Necrotic peripancreatic and periportal lymph nodes are present, measuring up to 2.6 cm in maximal dimension (black arrowheads, 2B). Nodular thickening of the left adrenal gland (black arrow, 2A) that demonstrated washout consistent with a lipid-poor adenoma.

Abdominal computed tomography (CT) showed a 5.9 ×5.6 cm solid mass in the tail of the pancreas interfacing with the spleen, stomach, colon, and peripancreatic and periportal lymph nodes. The left adrenal nodule had radiological characteristics of a lipid-poor adenoma (washout 69.4%) (Fig. 2).

## Treatment

**Figure 3.**
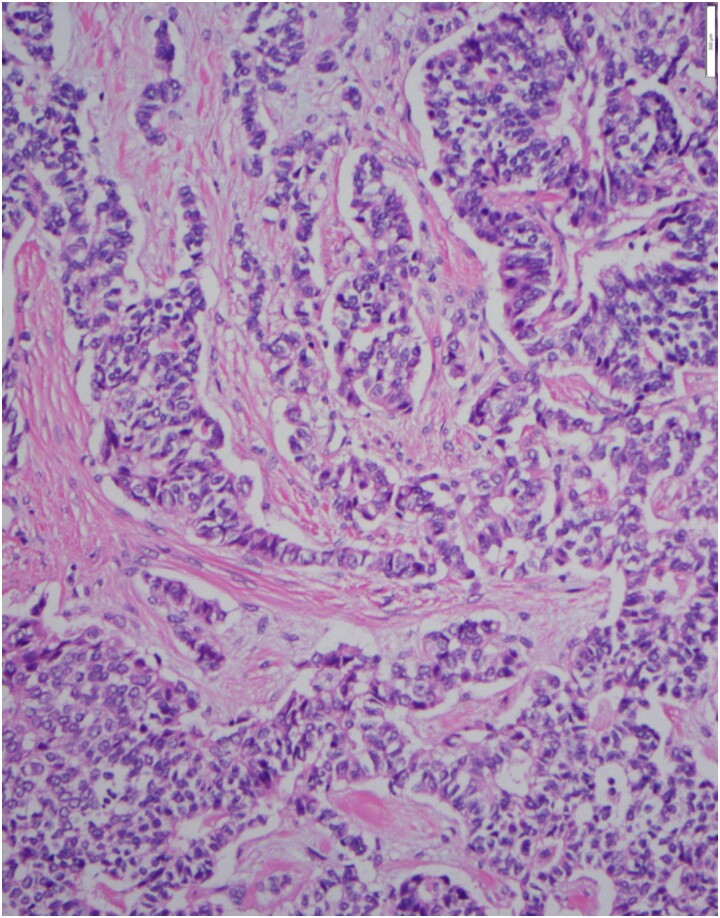
Neuroendocrine tumor with both solid and trabecular growth pattern in the background of fibrotic stroma (20×).

**Figure 4.**
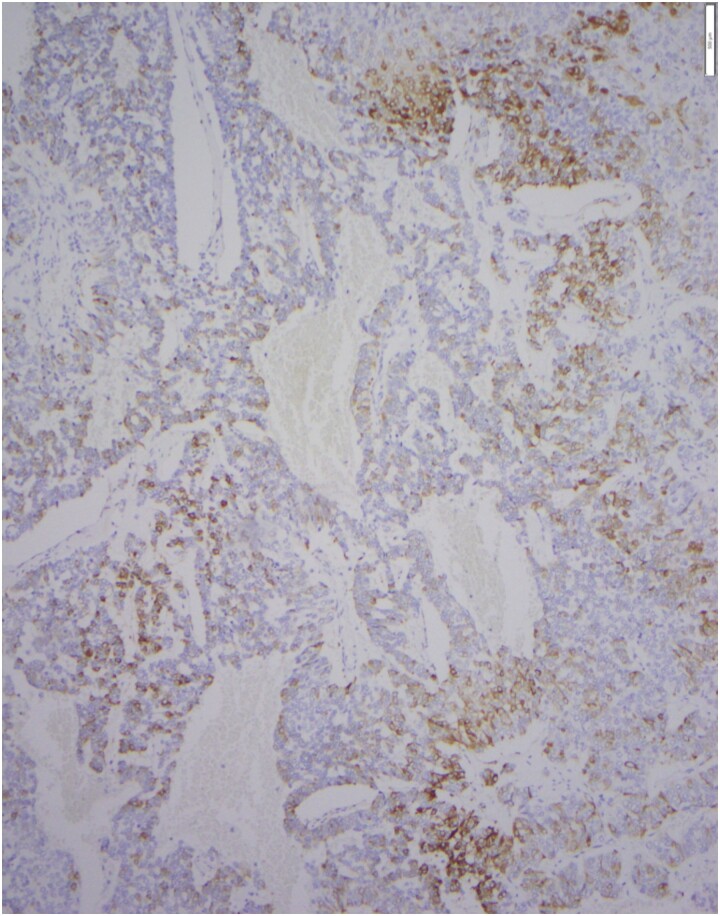
Immunostaining for ACTH shows patchy positive staining in tumor cells.

After CS workup concluded, the patient saw neurosurgery (who agreed with imaging surveillance of the pituitary macroadenoma), abdominal surgery, gastroenterology and medical oncology. Patient received ketoconazole 200 mg twice a day for 1 month prior to surgery. An endoscopic ultrasound with biopsy of the pancreatic mass confirmed a NEN. Surgery consisted of distal pancreatectomy, splenectomy en-bloc with transverse colon. Pathology showed a well-differentiated neuroendocrine tumor (Fig. 3), World Health Organization (WHO) grade 2, with invasion into the peripancreatic soft tissue, spleen, and the muscularis propria of the transverse colon, as well as lymphovascular and perineural invasion. Proliferation index Ki67 was 5% to 10%. Immunostaining was positive for cytokeratin, synaptophysin, and ACTH (Fig. 4). During surgery, duodenectomy was performed and pathology confirmed several duodenal NEN. Patient received stress dose hydrocortisone postoperatively and was discharged on oral hydrocortisone 20 mg in AM and 10 mg in PM.

At 3 months postoperatively, patient had weight loss (25 kg) and resolution of the leg edema, easy bruising, and rounded face. Blood pressure was normal on carvedilol alone. Her glycated hemoglobin (HbA1c) levels improved from 7.5% preoperatively to 6.4%, and metformin was eventually stopped.

In the first year postoperatively, early morning serum cortisol levels prior to taking hydrocortisone ranged between 1.1 and 2.2 μg/dL (30.4-60.7 nmol/L). Immediate postoperative ACTH levels were undetectable. The patient underwent a slow hydrocortisone taper and recovered cortisol production after 18 months. An ACTH stimulation test indicated baseline cortisol 8.4 μg/dL (231.8 nmol/L) and 60 minutes 19.8 μg/dL (546.5 nmol/L).

**Figure 5.**
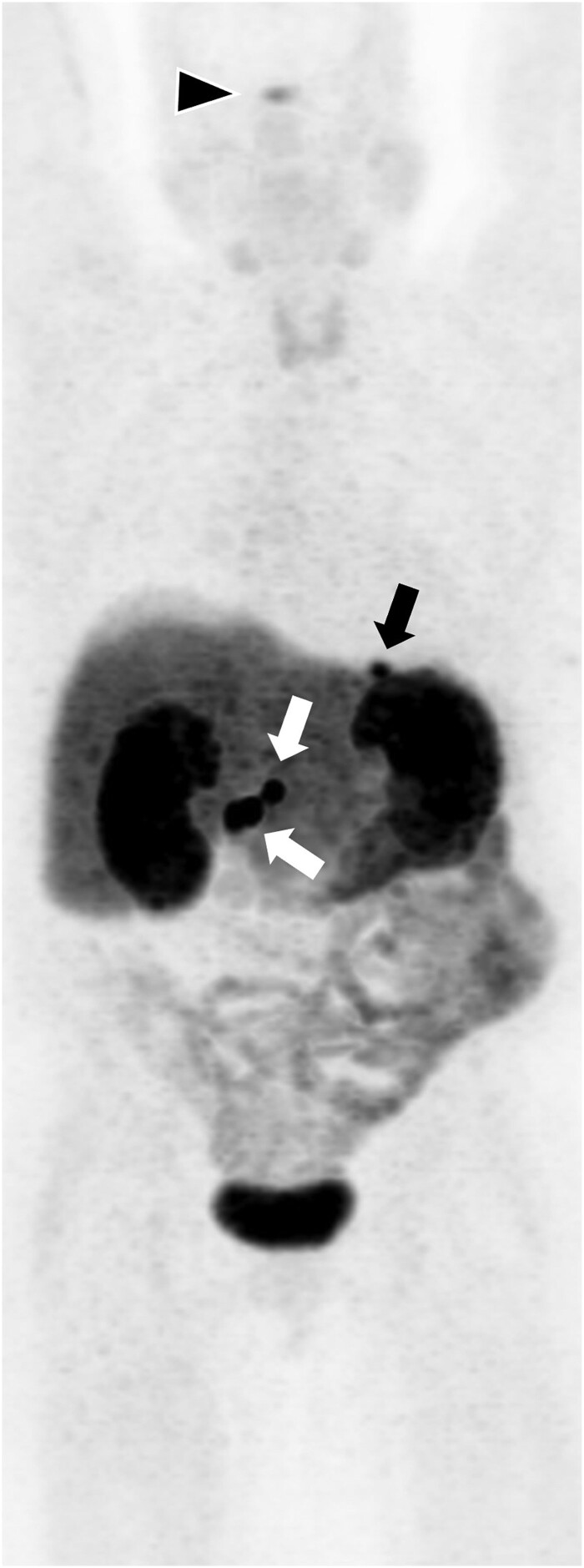
Maximal intensity projection image from the initial Ga-68 DOTA-TATE PET/CT obtained after pancreatic tumor resection demonstrates several somatostatin receptor–positive peripancreatic and porta hepatis lymph nodes (SUV max up to 27.1), measuring up to 1.3 cm in maximal dimension (white arrows). Increased tracer uptake is also seen in a small hepatogastric ligament lymph node (black arrow). Physiologic tracer uptake is seen in the pituitary gland (black arrowhead).

A DOTATATE positron emission tomography (PET)/CT scan 2 months postoperatively showed evidence of residual disease with multiple somatostatin receptor (SSR)-positive lymph nodes in the hepatic and pancreatic region (Fig. 5). The patient was started on lanreotide 120 mg subcutaneously monthly. Imaging 13 months after surgery showed disease progression with intensely SSR-positive lesions in the duodenum, pancreas, and 2 hepatic segments. The patient received peptide receptor radionucleotide therapy (PRRT), 4 doses of Lu-177 Octreotate every 2 months.

## Outcome and Follow-Up

Patient was followed for 3.5 years after pancreatic surgery without recurrence of hypercortisolism manifestations. Morning serum cortisol levels remained normal after stopping the hydrocortisone. ACTH levels normalized, then increased above normal along with the radiological disease progression. Most recent ACTH levels decreased from 149 to 84 pg/mL (from 32.8 to 18.5 pmol/L). Bedtime salivary cortisol levels were mostly normal or slightly elevated. Urinary cortisol levels were normal.

Abdominal imaging 3 months after PRRT indicated resolution or decrease in size of liver and duodenal lesions and no new SSR-avid lesions. Patient currently receives Octreotide 40 mg IM monthly. The pituitary adenoma remained stable or slightly smaller in size on annual scans without contact with the optic chiasm; annual evaluation for hypopituitarism were unremarkable. Bilateral adrenal enlargement was less pronounced, and the adrenal adenoma was also stable. Patient was referred for genetic testing for multiple endocrine neoplasia syndromes, which was not yet completed.

## Discussion

A case series (11 patients) and review of the literature (approximately 100) published in 2015 indicated that ACTH-secreting pNEN are usually large well-differentiated tumors with vascular and perineural invasion, similar with our case [4]. A recent systematic review (336 cases) published in English and Chinese language found that pNEN more commonly affected women (66.4%) and the mean age at presentation was 44.7 years. Most prevalent manifestations were hypokalemia (69.3%), hyperglycemia (63.2%), muscle weakness (60.1%), hypertension (56.4%), moon face (41.1%), edema (37.4%), and weight gain (23.3%) [3]. The most common pancreatic location was the tail (44.4%), followed by head (34.9%), and body (12.4%). Mean tumor size was 4.43 cm. Histologic confirmation of ACTH positivity was present in 84% cases. Metastases were reported in majority of patients, especially affecting the liver and lymph nodes. Follow-up ranged from 1 month to 20 years and 97 patient deaths were reported after a mean of 23 months [3].

Pituitary magnetic resonance imaging (MRI) is the first imaging test recommended after biochemical demonstration of ACTH-dependent CS. When tumor diameter is small (<6 or 10 mm), a bilateral inferior petrosal sinus sampling (BIPSS) is recommended to confirm the pituitary origin of ACTH [1]. In general, diagnosis of Cushing disease is presumed with a pituitary adenoma >10 mm, which prompts transsphenoidal surgery recommendation. In our case, pituitary MRI showed a macroadenoma; however, due to the rapidly evolving clinical scenario and significantly elevated ACTH and cortisol levels, we performed dynamic noninvasive testing with CRH. The test relies on presence of CRH receptors on pituitary but not ectopic ACTH-secreting tumors. After CRH stimulation, an ACTH increment from baseline by 31% to 50% is considered suggestive of Cushing disease. When CRH is not available, desmopressin testing can be used, as pituitary corticotroph tumors express vasopressin receptors. After high-dose dexamethasone (8 mg at night), 8 Am serum cortisol usually decreases to <5 μg/dL (<138 nmol/L) in pituitary adenomas, due to presence of glucocorticoid receptors on the tumor cells. However, this test has a low diagnostic precision for differentiation of ectopic CS [2]. All testing in CS has a probabilistic significance, with false positive and negative results both possible.

In patients with normal or very small pituitary tumors, when BIPSS is not available or when ectopic CS is strongly suspected, imaging of the neck, chest and abdomen are recommended [1, 5]. In our case, a large pancreatic mass was detected after dynamic testing indicated a high likelihood of ectopic CS. If transsphenoidal surgery had been recommended based on the pituitary MRI, hypercortisolism would not have resolved and delays in the pNEN treatment would have occurred. BIPSS was not necessary in our case due to convergent findings of the dynamic noninvasive hormonal testing, abdominal imaging, and finding of a neuroendocrine tumor through biopsy.

For ectopic CS, the typical scenario is of a male with hypokalemia and rapid progression of hypercortisolism manifestation over few weeks [6]. Historically, the majority of ectopic CS cases were due to small cell lung carcinoma, which was also associated with catabolic syndrome. However, the presentation of ectopic CS is more nuanced today, when NEN such as bronchial carcinoids and gastrointestinal/pancreatic NEN are increasingly encountered. Specifically for pNEN, age at diagnosis and gender predisposition are similar with pituitary Cushing's. Such cases are extremely rare and complex and require careful multidisciplinary management. Older studies indicated liver metastases were usually present at the time of diagnosis [7] and prognosis was guarded. The course was favorable in our case, with postoperative clinical and biochemical resolution of CS and control of residual SSR-positive lesions with somatostatin-based medical therapy and PRRT.

Regarding the pituitary lesion, the diagnosis of nonfunctional macroadenoma was based on the imaging appearance and hormone studies. Management of pituitary incidentalomas depends on tumor size, location, and imaging surveillance studies. Tumor growth can be expected in 24.1% of macroadenomas; decreased tumor size in 12.7%, and unchanged size (for up to 8 years) in 63.2% of macroincidentalomas [8]. Patients require careful surveillance for possible mass effects (including ophthalmological evaluation) and hypopituitarism testing [9].

## Learning Points

- Presentation with rapidly progressing hypokalemia, hyperglycemia, and myopathy raises suspicion of ectopic CS.
- Pancreatic ACTH-producing tumors are rare and affect predominantly middle-aged women.
- Coexistence of a pituitary and pancreatic lesion requires dynamic testing to determine the source of ACTH production.
- Confirmation of an ACTH-secreting pancreatic NEN relies on ACTH immunohistochemistry.
- Remission of hypercortisolism can occur in absence of extensive metastatic disease.

## Contributors

All authors made individual contributions to the authorship. C.B.: involved in data collection, manuscript writing, tables, and graphs production. A.G.I.: data collection, manuscript writing. J.W.A.: images and manuscript editing. Z.K.M.: pathology pictures and manuscript editing. All authors reviewed and approved the final draft.

## Data Availability

Not applicable to this article as no datasets were generated or analyzed during the current study.

## Abbreviations

ACTH: adrenocorticotropin hormone
BIPSS: bilateral inferior petrosal sinus sampling
CRH: corticotropin-releasing hormone
CS: Cushing syndrome
CT: computed tomography
MRI: magnetic resonance imaging
NEN: neuroendocrine neoplasm
pNEN: pancreatic neuroendocrine neoplasm
PRRT: peptide receptor radionucleotide therapy
SSR: somatostatin receptor
ULN: upper limit of normal
